# Guiding ethical principles in transplant candidate selection committees: A scoping review protocol

**DOI:** 10.1371/journal.pone.0324623

**Published:** 2025-06-02

**Authors:** Christopher H. Kim, Frank G. Lee, Tayyab S. Diwan, on behalf of the Transplant Ethics Research Lab

**Affiliations:** 1 Biomedical Ethics Research Program, Mayo Clinic, Rochester, Minnesota, United States of America; 2 Transplant Center, Mayo Clinic, Rochester, Minnesota, United States of America; Mayo Clinic College of Medicine and Science, UNITED STATES OF AMERICA

## Abstract

**Objective:**

The goal of this scoping review is to examine the published literature regarding ethical principles that guide solid-organ transplant candidate selection committees. The review will identify the available research and provide recommendations to selection committees.

**Introduction:**

Transplantation is a field where ethics is involved with almost every juncture of treatment. Organ selection, pathways to donation, organ allocation, and recipient selection are just a few of the myriads of processes that require ethical thoughtfulness. However, the ethical insights involved in the decision-making during these processes are not well elucidated in the literature. An application of an ethical framework(s) to this process could substantially improve the equality of the process.

**Inclusion criteria:**

This review will include studies focused on transplant candidate selection committees and/or the transplant candidate selection process. It will include full papers and abstracts published in English.

**Methods:**

PubMED, EMBASE, APA PsycInfo, SCOPUS, and CINAHL databases will be used for the literature search. Only articles published in English between the years 1960 and 2024 will be considered. The review will consist of title/abstract screening followed by full-text screening to determine which articles meet inclusion criteria. Each article will be reviewed by two independent reviewers, with a third stepping in to resolve conflicts in decision. After all articles have been screened, data will be extracted in duplicate from the selected included articles using a template in Covidence created by the study team. Results will then be summarized and presented in narrative summary.

**Discussion:**

An examination of the transplant selection process is due and warranted as selection carries significant weight in determining who receives an organ and, indeed, the stakes are life and death. Yet few studies exist on transplant selection. Ultimately, the problem with selection is how committees can further improve their promise to patients: are they making the right decision and how they define “the right decision.” Thus, it is critical to review selection committees for systematic bias and discrimination, especially in a process that depends heavily on individuals utilizing judgment-based reasoning, in order to improve selection and rid it of historic biases. This way, the transplant candidate selection process can foster fairness, thereby instilling increased public trust.

## Introduction

Much of transplantation is well-defined, algorithmic, and automatic (e.g., MELD prioritization in liver and composite allocation scoring for lung); however, the transplant selection process is uniquely human [1,2]. In the selection process, a committee of well-intentioned individuals--typically physicians, nurses, social workers--must decide on the allocation of organs. Because the demand for this life saving treatment far outweighs the number of organs available, the decision is placed upon the selection committee to decide who is eligible to receive an organ transplant. This process involves objective, impartial, and measurable data, such as condition of disease or medical urgency. However, unquantifiable, subjective factors may be considered, varying between and amongst transplant centers. Not only is this information complicated, but committee members’ interpretations of such information differ based on their respective background, beliefs, past experiences, morals, and what they may consider reasonable. Clear examples include amount of existing social support, a candidate’s briefest or longest period of sobriety or abstinence, extent of insight into their disease, and eagerness, endurance, and willingness to adhere to the entirety of transplant treatment. Hidden or subconscious examples may be social worth, past transgressions of missed appointments or microaggressions, race, gender, and socioeconomic status. In addition to the variations amongst committee members, selection committees between transplant centers also vary, from practice patterns to performance metrics to idiosyncratic cultural differences. A thoughtful examination of the transplant selection process, from its historical origins to now, could reveal interesting insights into its future.

For difficult problems like these – in which there exists no clear-cut answer, where one solution may favor one individual while disadvantaging another – turning to an ethical framework model can help provide clarity and definition. Transplant selection is a “wicked problem” – coined by Rittel and Weber in 1973 to describe such challenging problems like this, usually in difficult policy-making or large organizational models that are burdened by complexities [3,4]. A single adjustment intended to improve and benefit some can have downstream consequences that are hidden, delayed, and marginalize others. ***The objective of this project is to determine, via systematic review, ethical principles guiding transplant candidate selection committees in the candidate selection process in transplantation****.* A scoping review of this subject will hopefully demonstrate the gaps that are present in the understanding of the immense ethical challenges that exist in candidate patient selection.

### Review question

What are the ethical principles guiding transplant candidate selection committees in determining whether or not a patient should be eligible to be listed for a solid-organ transplant?

### Aims

1. Describe the current state of transplant candidate selection committees.
2. Categorize different types of ethical principles guiding transplant candidate selection committees.
3. Identify gaps in the research literature on ethical guidance for transplant selection committees.

## Methods and analysis

### Scoping review protocol design

The scoping review will be conducted following the PRISMA guidelines for scoping reviews along with the updated explanation statement [5,6]. These guidelines will allow for the development of a comprehensive protocol to search for literature related to transplant selection committees and the candidate selection process. The focus being on identifying gaps in the existing literature and then synthesizing what the gaps represent into a proposition of an ethical framework and decision-making design that would work best for transplant selection committees. The justification for a scoping review and not a systematic review was that we aimed to broadly map and understand the broad scope of the literature on this topic. This design is akin to what Arksey and O’Malley outline as their 4^th^ reason for a scoping review: “to identify gaps in the evidence base…” and to “summarize and disseminate research findings” for the purpose of being able to draw conclusions from the review. But as Arksey and O’Malley point out, it is not to distinguish poor quality literature from others since the review is not designed to make this assessment [7].

This study will utilize the 5-stage framework outlined by Arksey and O’Malley: Stage 1 – identifying the research question; Stage 2 – identifying relevant studies; Stage 3 – study selection; Stage 4 – charting the data; and Stage 5 – collating, summarizing and reporting the results.

#### Stage 1 – Identifying the research question.

The main research question focuses on determining the ethical principles which are involved in candidate selection during the transplant process. The preliminary questions which will act as a guide to assist in the review are as follows:

1. What ethical principles are currently employed in the transplant candidate selection process?
2. What ethical principles are involved specifically in the decision-making process in transplant candidate selection?
3. What gaps are evident in the literature pertaining to the transplant candidate selection committee process?
4. Are there ethical frameworks that can be established to improve the transplant candidate selection process for potential transplant candidates?

#### Stage 2 – Identifying relevant studies.

A literature search will be conducted to identify relevant studies between the years 1960–2024 focused on transplant selection committees and transplant candidates selection. Due to linguistic limitations of the research team and ability to pay for translation services, the search was limited to only those published in English. We acknowledge that this may pose a potential risk of bias. The following databases were searched: PubMED, EMBASE, APA PsycInfo, SCOPUS, and CINAHL. Keywords and index terms were adapted to match conventions of various electronic databases. Search strings will be modified to fit the syntax of the database that it is used in. Librarians developed a systematic search strategy and completed the search on July 1, 2024. The search strings used for each database are shown in Table 1.

**Table 1:** Search strategies for databases.

PubMed search strategy	
#	Search Query
#1	transplant*[Title] OR “Transplant Recipients”[Mesh]
#2	“selection committee”[Title/Abstract:~2] OR “selection process”[Title/Abstract:~2] OR “selection procedure”[Title/Abstract:~2] OR “selection committees”[Title/Abstract:~2] OR “selection processes”[Title/Abstract:~2] OR “selection procedures”[Title/Abstract:~2] OR “recipient selection”[Title/Abstract:~2] OR “transplant selection”[Title/Abstract:~2]
#3	#1 AND #2
#4	limit to English
EMBASE (Ovid platform, 1974–2024 June 14) search strategy	
#	Search Query
#1	“transplant*”.m_titl.
#2	((selection adj2 committee*) or (selection adj2 process*) or (selection adj2 procedur*) or (recipient adj2 selection) or (transplant adj2 selection)).ti. or ((selection adj2 committee*) or (selection adj2 process*) or (selection adj2 procedur*) or (recipient adj2 selection) or (transplant adj2 selection)).ab.
#3	1 and 2
#4	limit 3 to english language
APA PsycInfo (Ovid platform) search strategy	
#	Search Query
#1	“transplant*”.m_titl.
#2	((selection adj2 committee*) or (selection adj2 process*) or (selection adj2 procedur*) or (recipient adj2 selection) or (transplant adj2 selection)).ti. or ((selection adj2 committee*) or (selection adj2 process*) or (selection adj2 procedur*) or (recipient adj2 selection) or (transplant adj2 selection)).ab.
#3	1 and 2
#4	Limit 3 to english language
Scopus search strategy	
#	Search Query
#1	TITLE (transplant*)
#2	TITLE-ABS-KEY (selection W/2 committee) OR TITLE-ABS-KEY (selection W/2 process) OR TITLE-ABS-KEY (selection W/2 procedure) OR TITLE-ABS-KEY (selection W/2 committees) OR TITLE-ABS-KEY (selection W/2 processes) OR TITLE-ABS-KEY (selection W/2 procedures) OR TITLE-ABS-KEY (recipient W/2 selection) OR TITLE-ABS-KEY (transplant W/2 selection)
#3	1 AND 2
#4	LIMIT-TO (LANGUAGE, “English”)
CINAHL (EBSCO platform) search strategy	
#	Search Query
S1	TI transplant* OR MH “Transplant Recipients”
S2	(selection N2 committee*) OR (selection N2 process*) OR (selection N2 procedur*) OR (recipient N2 selection) OR (transplant N2 selection)
S3	S1 AND S2.
S4	S3 Narrow by Language: - English.

#### Stage 3 – Study selection.

Studies will be imported from the scholarly databases into Covidence and will undergo two levels of screening. The first level of screening will be of title/abstracts by two independent reviewers. In this level of screening, duplicates will be identified and deleted. The second level of screening will be a full-text review also by two independent reviewers. If a conflict arises by two reviewers, the principal investigator will attempt to resolve the issue, and if the conflict persists, the principal investigator will have the final decision-making power.

Inclusion criteria will be the following: 1) the study is about solid-organ transplant candidate selection committees and/or the solid-organ transplant candidate selection process; 2) the paper is either a full paper, which includes but is not limited to original articles, editorials, commentary, and/or an abstract presented at a national or international meeting; 3) the written work is published in English. Exclusion criteria will include 1) any study pertaining to transplantation in animals, 2) any articles not published in English, and 3) no mention of ethical principles or guidelines involved in the process. Studies will not be accessed for bias or for quality.

#### Stage 4 – Charting the data.

A standardized data extraction form will be developed to extract study characteristics. These characteristics included the following (in no specific order): ethics principles employed, guidelines utilized in selection process, gaps identified in selection process (including but not limited to specific ethical gaps), decision making processes utilized. The form will be utilized by all study members on five randomly selected studies. Reviewers will work independently to extract study details in duplicate. The principal investigator will review final data extraction and resolve any conflicts. The majority of themes will be created from the consistency in which they occur in the reviewed literature.

#### Stage 5 – Collating, summarizing and reporting the results.

Results of the literature search and review will be presented in a flow chart as per PRISMA guidelines [5]. The results will be presented in tables and in a narrative form. In addition to the summary of the results, conclusions driven from the review will also be discussed.

### Current status and timeline of the study

As of December 5, 2024, the study team has completed the first level of screening (title/abstract review) and has entered into the second level of screening (full-text review). The second level of screening is expected to be completed in April 2025, and the analysis of the data is expected to be completed in August 2025.

### Ethics

Due to the nature of this scoping review methodology, ethics approval is not necessary. There are no human subjects participating in this study.

### Dissemination

Results of this review will be disseminated at regional, national, and international conferences, as well as in peer-reviewed journals, both in the social science and medical community. Plain language documents will be utilized to assist in delivery of the results to patient stakeholders. The project will also be discussed on a broader scale with the transplant community in hopes of the possibility of development of a national trial to assess the current standards of the transplant candidate selection process and to determine quality measures that may be important.

## Discussion

An examination of the transplant selection process is due and warranted as selection carries significant weight in determining who receives an organ and, indeed, the stakes are life and death. Yet few studies exist on transplant selection. A study conducted by Volk et al. in 2011 observed four liver transplant centers’ selection committees for qualitative thematic analysis, highlighting teamwork, consensus, and respect [8]. Other studies, notably in liver transplant, showed systematic variability and bias in listing of Black patients, citing disparate psychosocial ratings when reviewers subjectively attempt to evaluate on the basis of and intersectionality of race and socioeconomic status [9–11]. Concerns of inter-center variability have also been raised. Many centers follow their own institutional practices, causing variation between centers, not to mention by each organ type. Selection is also a dynamic process with inherent procedural limitations. Consensus-first decision-making can be made ineffective when groups default to groupthink [12]. Furthermore, committee members often have a moral investment in these decisions, and when outcomes are not what one expects or severely disappoints, moral injury can occur [13].

Ultimately, the problem with selection is how committees can further improve their promise to patients: are they making the right decision and how they define “the right decision.” Systemic bias and discrimination may also exist within selection committees. Current news articles cite examples of patients being denied at one center and approved at another allegedly based on racially or socioeconomically charged reasons [14]. Thus, it is critical to review selection committee for systematic bias and discrimination, especially in a process that depends heavily on individuals utilizing judgment-based reasoning, in order to improve selection and rid it of historic biases [8,15]. This way, the transplant candidate selection process can foster fairness, thereby instilling increased public trust.

## Conclusion

The candidate selection process, a process that every patient considering transplant must undergo, has not been rigorously studied since the inception of the “God Committee” in 1962. Current models of the transplant candidate selection committee have not been rigorously tested nor analyzed, simply passed down from that first selection committee. As seen through the “God Committee,” these models of selection are susceptible to human errors, biases, institutional discrimination, and variability. By critically evaluating this key step, the added attention and scrutiny will yield a better version regardless of what is tackled first. But utilizing an ethical framework is a good starting point. Transplant candidate selection committees owe it to their transplant patients to provide the most ethical and fair process, especially since donors – living and deceased – entrust them with their greatest gift.
